# Acculturation, Communication Competence, and Family Functioning in Mexican–American Mother–Daughter Dyads

**DOI:** 10.1007/s10903-021-01256-x

**Published:** 2021-08-07

**Authors:** Becky Marquez, Tanya Benitez, Zephon Lister

**Affiliations:** 1 Herbert Wertheim School of Public Health, University of California San Diego, La Jolla, CA USA; 2 School of Public Health, Brown University, Providence, RI USA; 3 Department of Counseling and Family Sciences, Loma Linda University, Loma Linda, CA USA

**Keywords:** Acculturation discrepancy, Communication, Family functioning, Mexican–American families

## Abstract

Little is known of how intergenerational acculturation discrepancy relates to communication skills differences that may influence relationship quality among parents and adult children. Mexican–American mother–daughter dyads (n = 59) were studied using the Actor Partner Interdependence Model to examine dyadic associations of acculturation and communication competence with family functioning and mediation analysis to determine the indirect effect of acculturation discrepancy on family functioning through communication competence differences. Communication competence of mothers exerted significant actor and partner effects on daughter-perceived cohesion and closeness. Higher acculturation discrepancy predicted greater communication competence difference which in turn was associated with lower cohesion and closeness. There was a significant indirect effect of acculturation discrepancy on daughter-perceived cohesion through communication competence difference. Communication competence of mothers impacts their own as well as their daughters’ perceptions of dyad cohesion and closeness. Intergenerational discrepant acculturation contributes to discordant communication skills that impair family functioning, which has implications for psychological well-being.

## Introduction

Intergenerational acculturation discrepancy has been associated with lower family functioning in Latino families [1, 2] and is believed to increase risk of adverse health outcomes such as poor mental health in youth [1, 3]. Acculturation discrepancy in immigrant families arises when parent and child adopt aspects of the mainstream culture such as language, attitudes, and behaviors at different rates, resulting in discordant cultures [4]. Parent–child communication may mediate the link between acculturation discrepancy and health risk factors in Latino families [5].

Communication is a means by which family members establish and maintain relationships [6]. Communication competence is important to effective communication (i.e., achieving desired intent or goal) [7]. Communication competence consists of skills, such as expressiveness, self-disclosure, empathy, and assertiveness, which guide interpersonal interactions that provide the basis for evaluation of relational quality. For example, interpersonal communication competence is positively associated with social support satisfaction [8]. Moreover, dyads with similarly high interpersonal communication competence report greater satisfaction with their relationship [9].

Currently, little is known of how acculturation relates to communication competence differences in parent–child dyads. Communication competence along with factors such as language preferences and norms of respect are influenced by cultural orientation. Traditionally, communication in Latino families is hierarchical and indirect [10]. Among Mexicans, conflict resolution styles are consistent with a collectivist-orientated culture in using more accommodative and collaborative approaches than Americans which are culturally more individually-oriented [11]. Cultural incompatibilities may develop when acculturated children communicate with parents with less deference and more assertiveness, which is more normative in the dominant culture of the US [10, 12].

Studies suggest that discrepant cultures may weaken the parent–child relationship [3, 13]. Generally, the parent–child relationship becomes more positive as adolescents become adults [14] possibly because of greater similarities associated with the life course [15] however this may be distinct for Latino families. Among Latinos, young adults report more intergenerational conflict than adolescents [16, 17], and there is a strong association between intergenerational acculturation discrepancy and conflict especially among women which may be due to gender role expectation [17]. The heightened association in women suggests a need for further study in mother–daughter dyads.

In Latino families, family functioning is linked to intergenerational conflict [16]. Family functioning refers to interactions and relationships among family members that reflect connectedness such as cohesion and emotional closeness [18]. Family functioning influences the association of acculturation discrepancy and mental health outcomes in Latino parents [19] and youth [1]. Understanding how intergenerational acculturation discrepancy relates to a more proximal factor like communication competence of parent and child and the subsequent impact on family functioning in Latinas (i.e., adult daughters) is especially important because those that report feeling close to family have lower risks for mental health disorders and suicidal ideation [20].

The purpose of this study is to investigate the association between acculturation, communication competence, and family functioning in Mexican–American mother–daughter dyads. We expect to find that acculturation is associated with dissimilar communication competencies. We hypothesize that acculturation and communication competence of mothers and daughters predict their own (actor effect) and each other’s (partner effect) perception of family functioning. We also hypothesize that greater differences in acculturation and communication competence are associated with lower family functioning and that acculturation discrepancy has an indirect effect on family functioning through communication competence difference between mothers and daughters.

## Methods

### Participants

A community sample of 59 adult mother–daughter dyads participated in a mixed method study examining communication and eating and physical activity behaviors in Mexican–American families with overweight or obesity in San Diego County. Participants were recruited via local media outlets (newspaper, radio, or web-based advertisements), community events such as health fairs or conferences, and flyers posted at university and community sites. Women were deemed eligible to participate via a telephone screener if they self-identified as Mexican or Mexican–American, were 18–65 years old, had a body mass index of 25–50 kg/m^2^, were English or Spanish language literate, and had a mother or daughter who also met the same eligibility criteria (e.g., both mother and daughter had overweight or obesity). Mother–daughter dyads attended an in-person visit, conducted by bilingual and bicultural research staff, where they provided written informed consent, had their height and weight objectively measured, and completed assessments. Study measures were available in English and Spanish. Translated measures underwent a back-translation process and were reviewed by a community engagement committee. Participants received a $30 incentive for their involvement in the study. The study was approved by the internal review board at the University of California San Diego.

### Measures

#### Demographic Information

Participants provided information on age, education, and employment. History in the US (e.g., nativity and generational status) and language use were also assessed.

#### Acculturation

Acculturation was measured using the Acculturation Scale for Mexican Americans-II (ARSMA) [21]. The measure consists of 30-items rated on a 5 point Likert scale (α = 0.74) and contains two subscales: (1) Anglo Orientation Scale (AOS), and (2) Mexican Orientation Scale (MOS). The acculturation score is calculated by subtracting the MOS score from AOS score, with higher values reflecting greater acculturation. Acculturation discrepancy was calculated by the sum of the absolute difference between mother and daughter AOS and MOS scores, with higher values reflecting greater acculturation difference between mother and daughter [19, 22].

#### Communication Competence

Communication competence was measured using the Interpersonal Communication Competence (ICC) Scale [7]. This multi-dimensional measure consists of 30-items that are rated on a 5 point Likert scale (α = 0.84). It assesses ten skill areas under the following constructs: Self-disclosure (opens up or reveals), Empathy (understands other’s perspective), Social relaxation (responds with comfort and non-apprehension), Assertiveness (stands up for self), Interaction management (negotiates and regulates), Altercentrism (attentiveness), Expressiveness (communicates feelings and thoughts), Supportiveness (warmth), Immediacy (approachability), and Environmental control (persuasiveness). The ICC scale is used as a comprehensive measure of communication competence as well as subscales of specific skills. Difference in dyadic communication competence was calculated by the absolute difference in score method.

#### Family Functioning

Dyad family functioning measured as cohesion and closeness were assessed separately. Cohesion was measured with a 4 item scale consisting of “I feel able to share my feelings with my mother/daughter”, “I feel that my mother/daughter cares about me”, “I feel a lot of trust in my mother/daughter”, and “My mother/daughter handles my emotions well”. Items were rated on a 5 point Likert scale ranging from never to always (α = 0.84). Closeness was measured with a single item as “How would you characterize your relationship with your mother/daughter?” Responses were rated on a 5 point Likert scale ranging from not at all close to extremely close.

### Analysis

Descriptive statistics summarized demographic information. Paired sample t-tests determined differences between mothers and daughters. Correlation analyses examined associations between acculturation, communication competence, and family functioning.

**Fig. 1:**
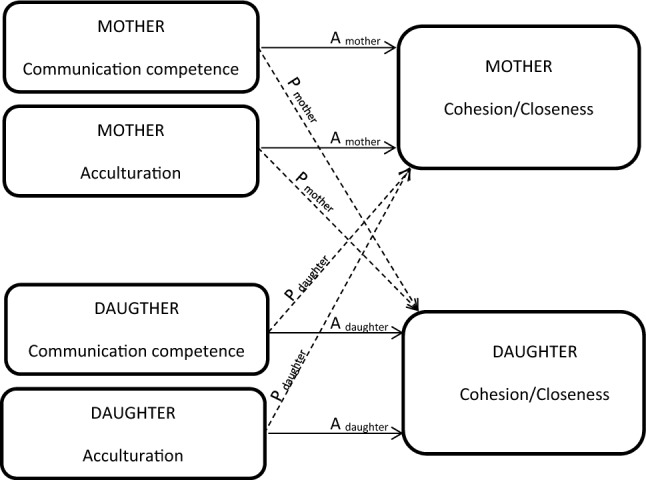
Conceptual diagram of APIM depicting actor (A) and partner (P) effects of acculturation and communication competence on family functioning in mother–daughter dyads

Analysis based on the Actor–Partner Independence Model [23] simultaneously tests the effect of a predictor from one member of the dyad on her own outcome (actor effect) as well as on the outcome for her mother or daughter (partner effect). APIM was performed using structural equation modeling with maximum likelihood estimation for distinguishable dyads [24]. Models included acculturation and communication competence as independent variables, age and education as covariates, and cohesion or closeness as the dependent variable (Fig. 1).

**Fig. 2:**
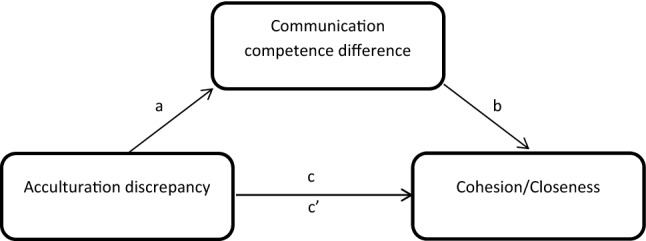
Conceptual diagram depicting the indirect effect of acculturation discrepancy on family functioning through communication competence difference in mother–daughter dyads

Mediation analysis was used to test the indirect of effect of acculturation discrepancy on cohesion or closeness through communication competence difference. Models controlled for age and education (Fig. 2). Bootstrap sampling was achieved using 10,000 iterations. An indirect effect was determined by a 95% bias-corrected bootstrap confidence interval (CI) that did not include zero. Analysis was conducted using macros in PROCESS version 3.3 [25].

## Results

### Participant Characteristics

Participant mothers and daughters were on average 49.8 ± 7.3 and 25.1 ± 5.9 years old, respectively (Table 1). Fewer mothers (47%) than daughters (84%) completed high school. About half of mothers (49%) and daughters (47%) had employment but more mothers were homemakers (40%) and more daughters were students (23%).

**Table 1:** Participant characteristics

	Mothers	Daughters
	(%)	(%)
Age (years)		
18–35	1.7	93.2
36–50	52.5	6.8
51–65	45.8	0.0
Education		
Less than high school graduate	52.5	15.3
High school graduate	33.9	79.7
College graduate	13.6	5.1
Employment		
Employed	49.1	47.5
Student	0.0	23.7
Homemaker	40.7	8.5
Retired	8.5	0.0
Unemployed	1.7	20.3
Generational status		
First generation	89.8	35.6
Second generation	8.5	54.2
Third generation	1.7	10.2
Language speak and read		
Spanish or more Spanish	74.5	13.5
Spanish and English equally	13.6	39.0
English or more English	11.9	47.5
ARSMA (mean ± standard deviation)	− 36.5 ± 19.6	− 16.3 ± 12.6

The majority of mothers were immigrants to the US (89%) and spoke and read predominately Spanish (74%) whereas the majority of daughters were born in the US (64%) and were bilingual (39%) or spoke and read predominately English (47%). Compared to mothers, daughters had higher ARSMA scores.

### Correlations Among Acculturation, Communication Competence, and Family Functioning

Acculturation of mothers was positively correlated with acculturation of adult daughters (Table 2). Communication competence or subscales were not significantly correlated between mothers and daughters (*p* > 0.05, Supplement). Cohesion and closeness were significantly correlated within and between mothers and daughters (Table 2).

**Table 2:** Acculturation, communication competence, and family functioning correlates in mother–daughter dyads

	1	2	3	4	5	6	7	8
1. Mother acculturation	–	0.195	0.099	0.132	0.505**	0.043	−0.112	−0.223
2. Mother communication competence		–	0.312*	0.314*	0.082	0.062	0.014	−0.070
3. Mother cohesion			–	0.689**	0.036	0.434**	0.345**	0.341**
4. Mother closeness				–	0.017	0.433	0.456**	0.494**
5. Daughter acculturation					–	−0.324*	−0.254	−0.264*
6. Daughter communication competence						–	0.555**	0.445**
7. Daughter cohesion							–	0.752**
8. Daughter closeness								–

^*^p < 0.05

^**^p < 0.01

Among mothers, acculturation was positively correlated with the Assertiveness subscale (α = 0.47; r = 0.31, *p* < 0.05). Among daughters, acculturation was negatively correlated with communication competence (Table 2), and subscales of Self-disclosure (α = 0.73; r = − 0.30, *p* < 0.05), Expressiveness (α = 0.46; r = − 0.32, *p* < 0.05), and Immediacy (α = 0.66; r = − 0.34, *p* < 0.01). Acculturation was also negatively correlated with perceived closeness (Table 2). Higher communication competence correlated with higher perceived cohesion and closeness.

### Interdependence of Acculturation, Communication Competence, and Family Functioning

There were no significant actor and partner effects of acculturation on family functioning (Tables 3, 4). There were significant actor and partner effects of communication competence on family functioning. Specifically, higher communication competence in dyads was associated with their own perception of cohesion (actor effect mother = 0.06, *p* = 0.01; actor effect daughter = 0.14, *p* < 0.01) and closeness (actor effect mother = 0.01, *p* = 0.01; actor effect daughter = 0.03, *p* < 0.01). Higher communication competence in mothers was associated with daughter’s perception of cohesion (partner effect = 0.12, *p* < 0.01) and closeness (partner effect = 0.03, *p* < 0.01). Daughters did not have significant partner effects on mothers (*p* > 0.05).

**Table 3:** Actor and partner effects of acculturation and communication competence on cohesion

Effect	Role	Estimate	95% CI	*p*
Intercept	Mother	2.304	−2.819–7.427	0.378
	Daughter	4.141	−0.391–8.673	0.073
Acculturation				
Actor	Mother	− 0.019	−0.064–0.027	0.422
Partner		0.050	−0.022–0.121	0.175
Actor	Daughter	− 0.001	−0.074–0.071	0.972
Partner		− 0.029	−0.073–0.014	0.190
Communication Competence				
Actor	Mother	0.067	0.016–0.118	**0.010**
Partner		0.124	0.063–0.1485	** < 0.001**
Actor	Daughter	0.143	0.083–0.203	** < 0.001**
Partner		0.002	−0.051–0.056	0.929

Bold indicates p value < 0.05

**Table 4:** Actor and partner effects of acculturation and communication competence on closeness

Effect	Role	Estimate	95% CI	*p*
Intercept	Mother	0.623	−0.692–1.938	0.353
	Daughter	0.853	−0.616–2.322	0.255
Acculturation				
Actor	Mother	−0.003	−0.015–0.009	0.610
Partner		0.010	−0.008–0.028	0.281
Actor	Daughter	−0.000	−0.023–0.023	0.984
Partner		−0.013	−0.027–0.001	0.066
Communication Competence				
Actor	Mother	0.017	0.004–0.031	**0.012**
Partner		0.032	0.016–0.048	** <0 .001**
Actor	Daughter	0.036	0.016–0.055	** < 0.001**
Partner		−0.003	−0.020–0.014	0.743

Bold indicates p value < 0.05

### Indirect Effect of Acculturation Discrepancy on Family Functioning Through Communication Competence

Higher acculturation discrepancy predicted greater difference in communication competence (a = 0.47, SE = 0.15, *p* < 0.01) between mother and daughter (Table 5). In turn, higher difference in communication competence was associated with lower mother-perceived closeness (b = − 0.02, SE = 0.01, *p* = 0.01) and daughter-perceived cohesion (b = − 0.10, SE = 0.04, *p* < 0.01). Total and direct effects were not significant (*p* > 0.05). There was a significant indirect effect of acculturation discrepancy on daughter-perceived cohesion through communication competence difference (ab = − 0.05, SE = 0.02, CI = − 0.122 to − 0.009).

**Table 5:** Indirect effect of acculturation on family functioning through communication competence

	a		b		c total effect		c′ direct effect		ab indirect effect	
Family functioning	*B (SE)*	*p*	*B (SE)*	*p*	*B (SE)*	*p*	*B (SE)*	*p*	*B (SE)*	95% CI
Mother										
Cohesion	0.472 (0.150)	**0.002**	− 0.043 (0.040)	0.293	− 0.043 (0.044)	0.334	− 0.022 (0.048)	0.640	− 0.020 (0.025)	− 0.075–0.025
Closeness	0.473 (0.148)	**0.002**	− 0.025 (0.010)	**0.019**	− 0.006 (0.012)	0.593	0.005 (0.012)	0.650	− 0.012.(008)	− 0.031–0.000
Daughter										
Cohesion	0.487 (0.150)	**0.002**	− 0.109 (0.040)	**0.008**	0.021 (0.046)	0.653	0.074 (0.048)	0.127	− 0.053 (0.029)	**− 0.122 to −0.009**
Closeness	0.487 (0.150)	**0.002**	− 0.017 (0.013)	0.182	− 0.001 (0.014)	0.893	0.006 (0.015)	0.671	− 0.008 (0.007)	− 0.025–0.001

Bold indicates p value < 0.05

## Discussion

The intergenerational acculturation gap has been proposed to lead to communication difficulties related to cultural conflict and emotional distancing in immigrant families [12]. The present study focused on the association of acculturation and communication skills with family functioning in Mexican–Americans. The results indicate that acculturation is related to different communication competencies and perceptions of family functioning in mothers and adult daughters.

Acculturation was associated with specific communication skills. Communication competencies have been found to be similar between mothers and their children [26], however, this did not apply to adult mother–daughter dyads in the current study, supporting the idea that dissimilar communication competencies may arise from different cultural orientations. Among mothers, high acculturation was associated with more assertive communication. Assertiveness, which includes direct expression of one’s viewpoint or standing up for oneself, has been described as a construct relevant to the Latino immigrant experience in the US [27]. Moreover, assertiveness was previously found to be higher in Puerto Rican women in the US with greater generational status, less sex-role traditionalism, and lower psychological distress symptomology [28]. Among daughters in the current study, high acculturation was associated with lower communication competence which suggests less effective management of interpersonal relationships. Daughters also communicated with less self-disclosure, expressiveness, and immediacy, which together indicate lower tendency for intimacy and openness during interpersonal interactions [7, 29].

Acculturation was associated with lower family functioning. Higher acculturated daughters perceived lower closeness with their mothers. Higher levels of family conflict and lower levels of family cohesion [30] or closeness [20] have been reported in US born Latinas compared to immigrant Latinas. Latinas report higher levels of familism, (i.e., value for family connectedness) compared to other groups and familism is associated with better psychological well-being indirectly through family closeness and support [31]. However, familism decreases with increased acculturation in Latinos [32]. Interestingly, there was significant agreement in reports of cohesion and closeness by mothers and daughters suggesting similar perspectives of relational quality.

Communication competence of mothers impact their own as well as their daughters’ perceptions of family functioning. Whereas mothers perceived greater family functioning when they themselves had higher communication competence, daughters perceived greater family functioning when they and their mothers had higher communication competence providing evidence for interdependence. Hence, daughters experience or view their relationship with their mother as more cohesive and close when mothers communicate with skills such as empathy, self-disclosure, expressiveness, and supportiveness. Despite the use of APIM to examine relationships among family members in various studies, it has been underused to examine acculturation and family outcomes in immigrant families. Although the current study did not find actor and partner effects of acculturation on family functioning, APIM was previously used to determine interdependent effects of acculturation and conflict in parent–adolescent Chinese–American dyads [33]. APIM is notably appropriate for studying adult parent–child dyads because a shift towards a more egalitarian relationship emerges that facilitates reciprocal effects on health attitudes and behaviors particularly among mothers and their adult children [34]. Furthermore, family communication is a significant predictor of health attitudes and behavior in these families.

A wider gap in acculturation may translate into greater differences in communication skills that in turn reduce perception of family functioning. There was a significant indirect effect of acculturation discrepancy on cohesion perceived by daughters. Dissimilar communication styles linked to cultural orientation was previously proposed to result in increased misunderstandings or conflicts [12]. Acculturation discrepancy has been associated with poorer parent–child communication [5], greater conflict [3, 13], and lower family functioning [1, 35] in Latino families. However, better parent–child communication buffers the negative effects of an acculturation gap in adolescents [36]. The present study suggests that greater difference in acculturation leads to incompatibilities in communicative approaches, which may worsen parent–child communication and foster emotional distancing between mothers and adult children.

This study has limitations to consider. The data are cross-sectional and causality cannot be determined. The study sample size is small and may be underpowered to detect some effects. The sample was not randomly selected and consisted of Mexican–American families from a US-Mexico border region and may not be representative of other Mexican–Americans or Latinos elsewhere in the US. Only adult mother–daughter dyads were examined and findings are not generalizable to other parent–child relationships (e.g., father–daughter). Moreover, it is possible that our findings were influenced by the weight status of our sample as the study only included families with overweight or obesity and excess weight has been previously associated with poorer family functioning [37]. Further, the study assessed the emotional bonding or connectedness aspect (i.e., family cohesion and closeness) of family functioning and did not include other aspects such as family communication and conflict [38].

## Contribution to the Literature

This study has several strengths. First, the study sample included young adult Latinas which are a largely understudied group despite being at high risk for psychological distress [39]. Also, finding factors associated with impaired family functioning is warranted given that Latinas who report feeling close to family have lower risks for mental health disorders and suicidal ideation [20]. Second, the APIM was used to examine intergenerational acculturation and communication, which is an appropriate analytical approach for determining mutual influences in families, and this is one of the few studies to do so. Third, mediation analysis was used to gain insight into the pathway by which acculturation differential leads to ineffective communication associated with reduced family functioning. Finally, this study focused on communication competence, which is a more proximal factor to adult parent–child communication and contributes to the understanding of how culturally influenced characteristics may underlie problematic interactions in immigrant families. Future studies would benefit from examining the relationship between communication styles and communication patterns or quality especially among adult parent–child dyads given that communication and interaction styles are the most commonly attributed source of relational conflict [40].

## Conclusion

The present study determined that differences in acculturation manifest in differences in communication skills which impact family functioning in Mexican–Americans. The results suggest that immigrant families with discrepant acculturation may experience impaired family functioning, such as decreased cohesion or closeness, which may be driven by discordant communication competencies. Identifying communication-specific mediators involved in the mechanism by which acculturation gap interferes with family processes is therefore important. Findings also further support the idea that working with immigrant families should involve addressing biculturalism and communication skills to facilitate family connectedness and psychological well-being [4, 12].
